# Endometrioid Immunohistochemical Prognostic Score Integrating SIRT1, HMGB1, Bcl-2, and Caspase-3 Expression in Endometrioid-Type Endometrial Cancer

**DOI:** 10.3390/diagnostics16172701

**Published:** 2026-08-24

**Authors:** Birant Boldan, Batuhan Kunt, Figen Efe Çamili, Atilla Kunt, Abdurrahman Yiğit, Gürhan Güney, Mine İslimye Taşkın, Gülay Turan, Yunus Emre Yörük, Selim Afsar

**Affiliations:** 1 Department of Obstetrics and Gynecology, Tepecik Training and Research Hospital, 35020 İzmir, Turkey; birantboldan@hotmail.com; 2 Department of Obstetrics and Gynecology, Faculty of Medicine, Balıkesir University, 10145 Balıkesir, Turkey; batuhan.kunt@hotmail.com (B.K.); efefigenefe@gmail.com (F.E.Ç.); guney.gurhan@mayo.edu (G.G.); minetaskin1302@yahoo.com.tr (M.İ.T.); 3 Department of Obstetrics and Gynecology, Bigadiç State Hospital, 10440 Balıkesir, Turkey; atillakunt1@gmail.com; 4 Department of Medical Oncology, Health Sciences University, Mersin State Hospital, 33210 Mersin, Turkey; dryigit@yahoo.com.tr; 5 Department of Pathology, Faculty of Medicine, Balıkesir University, 10145 Balıkesir, Turkey; gulaytr@yahoo.com.tr; 6 Information Processing Research and Application Center, Balıkesir University, 10145 Balıkesir, Turkey; yunus.yoruk@balikesir.edu.tr

**Keywords:** endometrial cancer, endometrioid carcinoma, immunohistochemistry, prognostic score, SIRT1, HMGB1

## Abstract

**Background/Objectives:** Prognostic stratification of stage I endometrioid-type endometrial cancer remains imperfect when based on clinicopathological variables alone. This retrospective study evaluated immunohistochemical markers reflecting stress adaptation, inflammatory signaling, autophagy regulation, and apoptotic balance, and developed an exploratory Endometrioid Immunohistochemical Prognostic Score (EIPS) based on SIRT1, HMGB1, Bcl-2, and Caspase-3 expression. **Methods:** Using surgical specimens from 139 patients with stage I endometrioid-type endometrial cancer, EIPS was calculated by assigning one point for high SIRT1, high HMGB1, high Caspase-3, and low Bcl-2 expression. Associations with overall survival and model discrimination were evaluated using Kaplan–Meier analysis, Cox regression, ridge-penalized multivariable modeling, and ROC/AUC analysis. **Results:** During a median follow-up of 1577 days, 12 deaths occurred. High SIRT1, HMGB1, and Caspase-3 expression were associated with poorer overall survival, while low Bcl-2 was retained as a biologically plausible adverse component. EIPS stratified patients into ordered low-, intermediate-, and high-risk groups with increasing event rates and showed higher apparent mortality discrimination than the predefined LVSI/grade-based clinicopathologic grouping (AUC = 0.857 vs. 0.756). IE-EIPS provided a modest additional apparent improvement in discrimination (AUC = 0.876). **Conclusions:** EIPS may offer prognostic stratification in stage I endometrioid-type endometrial cancer by integrating biologically complementary immunohistochemical markers.

## 1. Introduction

Endometrial cancer is the most common gynecologic malignancy in developed countries, and its incidence continues to rise, partly because of increasing life expectancy and the growing prevalence of obesity. In the United States, approximately 68,270 new cases and 14,450 deaths were projected for 2026, underscoring the growing clinical burden of the disease [1]. Although most patients are diagnosed at an early stage, outcomes remain heterogeneous. Conventional clinicopathological parameters, including tumor grade, depth of myometrial invasion, and lymphovascular space invasion (LVSI), remain central to contemporary guideline-based risk stratification and treatment planning, but they may not fully capture the biological diversity of endometrioid-type tumors [2]. Contemporary endometrial cancer practice increasingly incorporates molecular classification, including POLE-mutated, mismatch repair-deficient, p53-abnormal, and no-specific-molecular-profile subgroups, because these categories refine prognosis and may influence adjuvant treatment decisions beyond anatomic stage and histologic grade [3,4].

Sirtuin 1 (SIRT1) is a nicotinamide adenine dinucleotide (NAD^+^)-dependent deacetylase involved in DNA repair, cellular stress response, inflammation, metabolism, and apoptosis. In endometrial carcinoma, SIRT1 expression has been reported to be higher than in non-neoplastic endometrium and has been implicated in tumor growth, autophagy, and resistance to cisplatin and paclitaxel [5,6,7,8]. These findings suggest that SIRT1 may reflect stress-adaptive tumor biology and could contribute to prognostic heterogeneity in endometrial cancer.

High mobility group box 1 (HMGB1) is a non-histone nuclear protein involved in chromatin organization and transcriptional regulation. Under cellular stress, HMGB1 can be released extracellularly and act as a damage-associated molecular pattern, promoting inflammatory signaling, autophagy, invasion, angiogenesis, and therapy resistance [9,10]. In endometrial carcinoma, HMGB1-related mechanisms have been associated with chemotherapy-related autophagy and epithelial-to-mesenchymal transition, although the direction and magnitude of its effects may vary by experimental context [11,12].

Autophagy and apoptosis are closely linked processes in tumor adaptation and treatment response. Beclin-1 is a key autophagy regulator with a context-dependent role in cancer: it may suppress early tumorigenesis by maintaining cellular homeostasis, yet support survival of established tumors under metabolic or therapeutic stress [13,14]. In endometrial adenocarcinoma, high Beclin-1 expression has been associated with poorer prognosis, highlighting the need to evaluate autophagy-related markers within the broader biological context of each tumor [15,16].

Bcl-2 and Caspase-3 represent opposing but complementary aspects of apoptotic regulation. Reduced Bcl-2 expression and altered Bcl-2/Bax balance have been linked to adverse clinicopathological features in endometrial carcinoma, whereas Caspase-3 reflects activation of the execution phase of apoptosis [17,18,19]. Although Caspase-3 is typically considered a marker of cell death, increased tumor expression may also indicate high apoptotic turnover and aggressive tumor biology, with shorter survival reported in some endometrial cancer cohorts [18,19].

This study focused on pure endometrioid-type endometrial carcinoma, the most common histologic subtype, to reduce histologic heterogeneity within stage I disease. The selected markers were chosen to sample complementary pathway-related signals relevant to tumor behavior: SIRT1 for stress adaptation, HMGB1 for inflammatory and damage-associated signaling, Beclin-1 for autophagy regulation, Bcl-2 for anti-apoptotic signaling, and Caspase-3 for execution-phase apoptosis.

Together, these markers represent biologically interconnected pathways involving stress adaptation, inflammatory/autophagy signaling, and apoptosis-related regulation. We hypothesized that their combined assessment could improve prognostic stratification beyond conventional clinicopathological factors. Therefore, this retrospective cohort study evaluated SIRT1, HMGB1, Beclin-1, Bcl-2, and Caspase-3 expression in endometrioid-type endometrial cancer and developed the immunohistochemistry-based Endometrioid Immunohistochemical Prognostic Score (EIPS) using the markers most closely associated with overall survival.

## 2. Materials and Methods

### 2.1. Study Design and Patients

#### 2.1.1. Study Setting and Population

This single-center retrospective cohort study included patients diagnosed with endometrioid-type endometrial adenocarcinoma at the Department of Obstetrics and Gynecology, Balıkesir University Faculty of Medicine Hospital, between 1 January 2019 and 27 May 2022. Eligible patients were those with uterus-confined FIGO 2009 stage IA and IB pure endometrioid-type endometrial carcinoma and accessible follow-up data. Non-endometrioid and mixed histologic subtypes were excluded because they differ substantially from endometrioid carcinoma in tumor biology, molecular classification, therapeutic approach, and prognosis [2,3,4]. The study was approved by the Ethics Committee of Balıkesir University (Approval No. 2025/50; 4 February 2025), and the requirement for informed consent was waived because of the retrospective design and anonymized data.

#### 2.1.2. Exclusion Criteria

Patients were excluded if they had received neoadjuvant chemotherapy (n = 2), radiotherapy (n = 1), or hormonal therapy before surgery (n = 4). Additional exclusions were insufficient or unsuitable tissue samples for immunohistochemical analysis (n = 3), incomplete clinical or pathological data (n = 4), or concurrent or prior malignancy (n = 2). Patients with inflammatory disease (n = 4), infectious disease (n = 5), or autoimmune disease (n = 5) were also excluded because these conditions could potentially affect biomarker expression and systemic inflammatory indices.

#### 2.1.3. Staging and Final Cohort

FIGO staging was reviewed and reported according to the FIGO 2009 criteria. After application of the eligibility criteria, the final analysis included 139 patients.

#### 2.1.4. Follow-Up and Outcome Assessment

Patients were followed at Balıkesir University Hospital every 3 months for the first 2 years and every 6 months thereafter. Recurrences were confirmed by histological or cytological examination. Survival data were obtained from the Balıkesir University Miamed Hospital Data System and verified by telephone contact with patients or their relatives when necessary. Overall survival was calculated from the date of surgery to death from any cause or the last documented contact. Patients who were alive at the last documented contact were censored at that date. The final follow-up date was defined individually as the date of death or the most recent documented clinical or telephone contact. The administrative data-lock date for the cohort was 29 May 2026; patients without a death event before that date were censored at their last documented contact.

### 2.2. Immunohistochemistry and Scoring

#### 2.2.1. Tissue Preparation and Antibody Panel

Immunohistochemistry was performed on formalin-fixed, paraffin-embedded surgical specimens. Representative tumor blocks were selected after hematoxylin and eosin review. Sections were stained for SIRT1, HMGB1, Beclin-1, Bcl-2, and Caspase-3 using Santa Cruz Biotechnology (Dallas, TX, USA) antibodies: SIRT1 clone B-7, sc-74465; HMGB1 clone B-5, sc-518194; Beclin-1 clone E-8, sc-48341; Bcl-2 clone C-2, sc-7382; and Caspase-3 clone 4.1.18, sc-65497. The manufacturer-recommended dilution range was 1:50–1:500 for all primary antibodies, with 1:50 used as the starting dilution. Incubation was performed overnight at 4 °C for all markers.

#### 2.2.2. Staining Procedure

Sections were deparaffinized, rehydrated through graded ethanol, and subjected to heat-induced epitope retrieval using laboratory-validated conditions for each antibody; Bcl-2 retrieval used 10 mM sodium citrate buffer (pH 6.0). Endogenous peroxidase and nonspecific background staining were blocked, after which sections were incubated with primary antibodies under the conditions described above, followed by a horseradish peroxidase-conjugated secondary antibody system. Immunoreactivity was visualized with diaminobenzidine, counterstained with hematoxylin, dehydrated, mounted, and coverslipped. Internal tissue controls, positive-control tissues selected according to the manufacturer’s datasheets and laboratory-validated control protocols for each antibody, and negative controls with omission of the primary antibody were included where appropriate.

The marker-specific antibody dilutions and positive tissue controls were as follows: SIRT1, clone B-7, sc-74465, dilution 1:50, human breast carcinoma tissue; HMGB1, clone B-5, sc-518194, dilution 1:50, human tonsil tissue; Beclin-1, clone E-8, sc-48341, dilution 1:50, human breast carcinoma tissue; Bcl-2, clone C-2, sc-7382, dilution 1:50, human tonsil tissue; and Caspase-3, clone 4.1.18, sc-65497, dilution 1:50, human tonsil tissue. These antibody dilutions and control tissues were selected according to the corresponding manufacturer datasheets and processed in parallel with study specimens.

#### 2.2.3. Scoring and Analysis

Staining was evaluated in representative tumor areas by an experienced pathologist blinded to survival status, follow-up, recurrence, clinicopathological risk grouping, and statistical outcomes; slides were coded and clinical data anonymized before review. Staining intensity was scored as 0 = none, 1 = weak, 2 = moderate, and 3 = intense. Scores 0–1 were classified as low expression and scores 2–3 as high expression. Representative examples of marker-specific immunostaining intensity are provided in Supplementary Figures S1–S15. To assess reproducibility, all slides were independently reviewed by two pathologists using the same predefined 0–3 intensity scale after joint calibration on representative training cases. Discrepant or borderline scores were resolved by joint microscopic review and consensus before final data entry. Formal interobserver agreement statistics were not available for the original dataset and therefore could not be reported retrospectively as a kappa value. Marker localization was assessed according to expected patterns: nuclear/cytoplasmic for SIRT1 and HMGB1, cytoplasmic for Beclin-1 and Bcl-2, and cytoplasmic and/or nuclear for Caspase-3.

### 2.3. Statistical Analyses

The primary objective of the statistical analysis was to evaluate whether an immunohistochemistry-based Endometrioid Immunohistochemical Prognostic Score (EIPS) improves survival risk stratification in endometrioid-type endometrial cancer. Overall survival was defined as the time from surgery to death from any cause or last follow-up, and patients who were alive at the last follow-up were censored. The Inflammatory-Enhanced Endometrioid Prognostic Score (IE-EIPS) was evaluated as an ancillary exploratory model rather than as the primary prognostic model.

Continuous variables were summarized as mean ± standard deviation or median with interquartile range, according to their distribution, whereas categorical variables were summarized as frequencies and percentages. Continuous variables were compared using one-way ANOVA or Kruskal–Wallis tests, followed by appropriate post hoc comparisons when required. Categorical variables were compared using chi-square or Fisher exact tests, as appropriate.

Clinical grouping was defined a priori using lymphovascular space invasion and tumor grade. Patients were categorized into three mutually exclusive groups: Grade 1–2 LVSI-negative, Grade 1–2 LVSI-positive, and Grade 3. Grade 1–2 LVSI-negative tumors served as the reference group. To avoid overlap, the hierarchy used for this clinicopathological grouping was as follows: any grade 3 tumor was assigned to the grade 3 group regardless of LVSI status; among remaining grade 1–2 tumors, LVSI-positive cases were assigned to the LVSI-positive group, and LVSI-negative cases were assigned to the LVSI-negative reference group. Full ESGO/ESTRO/ESP risk-group classification was not used as the comparator because this retrospective cohort lacked several variables required for contemporary guideline-based risk assignment, particularly molecular subtype data such as POLE mutation status, mismatch repair status, and p53 status; therefore, the comparator was limited to an available, transparent LVSI/grade-based clinical grouping.

In the absence of genomic classification, the predefined hierarchical clinical groups were interpreted as clinicopathologic comparators rather than as formal molecular ESGO/ESTRO/ESP risk groups [2,3,4]. Grade 3 tumors were classified as a separate adverse group regardless of LVSI status; among grade 1–2 tumors, cases were further stratified according to LVSI status. Accordingly, LVSI-negative grade 1–2 tumors most closely approximated a lower-risk population and may overlap with Mayo low-risk criteria when additional requirements regarding myometrial invasion and tumor size are fulfilled [20]. LVSI-positive grade 1–2 tumors represented an adverse non-low-risk subgroup, broadly corresponding to high-intermediate risk when LVSI is substantial or diffuse [2]. Grade 3 tumors fell outside Mayo low-risk criteria and corresponded to intermediate or high-intermediate postoperative risk depending on stage and LVSI extent [2,20]. This clinicopathologic framework was used to provide an interpretable comparator for EIPS in this retrospective cohort, while acknowledging that it does not replace contemporary molecular risk stratification [2,3,4].

The five-marker IHC panel was selected a priori to capture complementary pathway-related signals that are feasible to assess in routine pathology material: SIRT1 for stress-adaptation, HMGB1 for inflammatory and damage-associated signaling, Beclin-1 for autophagy regulation, Bcl-2 for anti-apoptotic/differentiation-associated signaling, and Caspase-3 for execution-phase apoptosis. Marker selection was therefore based on biological plausibility and prior literature rather than data-driven optimization, and the final EIPS retained only the markers that provided the clearest adverse-risk signal in this cohort. Ki-67 was not included because standardized Ki-67 staining and scoring data were not available across the retrospective cohort.

SIRT1, HMGB1, Beclin-1, Bcl-2, and Caspase-3 expression levels were evaluated according to immunohistochemical intensity scores. For survival modeling, biomarkers were dichotomized a priori into low-expression (score 0–1) and high-expression (score 2–3) groups. The EIPS ranging from 0 to 4 was then generated by assigning one point for each adverse immunohistochemical feature: high SIRT1 expression, high HMGB1 expression, high Caspase-3 expression, and low Bcl-2 expression. Patients were classified as low risk (EIPS 0–1), intermediate risk (EIPS 2), or high risk (EIPS 3–4).

The IE-EIPS was calculated as an ancillary exploratory score by combining EIPS with clinical group, systemic immune-inflammation index (SII = platelet count × neutrophil count/lymphocyte count), and neutrophil-to-lymphocyte ratio (NLR = neutrophil count/lymphocyte count). SII and NLR were selected as exploratory routine blood-based inflammatory indices because prior endometrial cancer studies have reported their prognostic relevance [21,22]. SII was calculated from complete blood count parameters using the same laboratory reporting scale for platelet, neutrophil, and lymphocyte counts; therefore, reported SII values reflect this institutional unit scale and should be interpreted comparatively within the cohort. One additional point was assigned for each of the following: adverse clinical grouping, defined as LVSI-positive or grade 3 disease; SII at or above the cohort median; and NLR at or above the cohort median. These points were added to the EIPS to generate a total IE-EIPS ranging from 0 to 7. IE-EIPS risk groups were categorized as low risk (0–2), intermediate risk (3–4), and high risk (5–7). Kaplan–Meier curves were generated for clinical groups, individual biomarkers, EIPS risk groups, and IE-EIPS risk groups, and survival differences were evaluated using the log-rank test.

Univariate Cox proportional hazards regression was used to assess individual clinical and biomarker predictors of mortality. Because the number of survival events was limited, ridge-penalized multivariable Cox regression was used to reduce the risk of model overfitting, and multivariable findings were interpreted cautiously as exploratory. To align the multivariable analysis with the intended clinical use of EIPS, the penalized Cox model was also fitted using EIPS as categorical risk groups, with low-risk EIPS (0–1) as the reference and intermediate-risk EIPS (2) and high-risk EIPS (3–4) entered as indicator variables. Results were reported as hazard ratios with 95% confidence intervals. Kaplan–Meier and Cox regression analyses were used as the primary time-to-event methods. ROC/AUC analysis was performed as an exploratory discrimination analysis based on binary mortality status at the administrative censoring date, and the clinical grouping, EIPS, and IE-EIPS models were compared. EIPS was treated as the main prognostic model, whereas IE-EIPS was interpreted as an ancillary exploratory model. Bootstrap-based comparisons were used for AUC differences where applicable, and internally corrected AUC estimates were interpreted as exploratory validation metrics rather than definitive optimism-corrected performance estimates, consistent with recommendations for transparent reporting and cautious validation of prediction models [23,24].

Complete-case analysis was used for the primary variables. Statistical significance was defined as a two-sided *p*-value below 0.05. Exploratory analyses, including IE-EIPS, ROC/AUC comparisons, internal validation metrics, and multivariable modeling, were interpreted cautiously because only 12 deaths occurred during follow-up. Internal validation procedures, including bootstrap correction and 5-fold cross-validation, were used only to assess exploratory stability and were not considered a substitute for external validation.

Analyses were performed using Jamovi statistical software (version 2.3.21; Sydney, Australia) and R software (version 4.5.3; R Foundation for Statistical Computing, Vienna, Austria). Survival analyses were performed using the survival and survminer packages. Penalized regression analyses were performed in R using the glmnet package. Cross-validation procedures were performed using the tidymodels framework. Receiver operating characteristic analyses and AUC-ROC estimates were obtained using the pROC package. The optimal probability threshold for classification was selected using Youden’s index. For forest plot generation, the forestplot package was used. Decision curve analysis was performed using the dcurves and DecisionCurve packages. Visualization was performed using Python software (version 3.10; Python Software Foundation, Wilmington, DE, USA) with the matplotlib library version 3.11.0.

## 3. Results

### 3.1. Patient Cohort and Survival Outcomes

The final cohort comprised 139 patients with endometrioid-type endometrial carcinoma, with baseline demographic, clinicopathological, immunohistochemical, inflammatory, and outcome characteristics summarized in Table 1. Over a median follow-up of 1577 days, 12 deaths were recorded, yielding an overall event rate of 8.6%. When patients were stratified according to lymphovascular space invasion (LVSI) status and tumor grade, clinical grouping was significantly associated with overall survival. The groups were mutually exclusive by design: grade 3 tumors were assigned to the grade 3 group irrespective of LVSI status, while grade 1–2 tumors were separated according to LVSI status. Grade 3 tumors represented 19/139 (13.7%) patients in the cohort. Histologic grade and FIGO stage reflected different clinicopathological dimensions; grade 3 tumors were present in both stage IA (n = 9) and stage IB (n = 10) disease, while the remaining 120 patients had grade 1 or grade 2 tumors.

**Figure 1:**
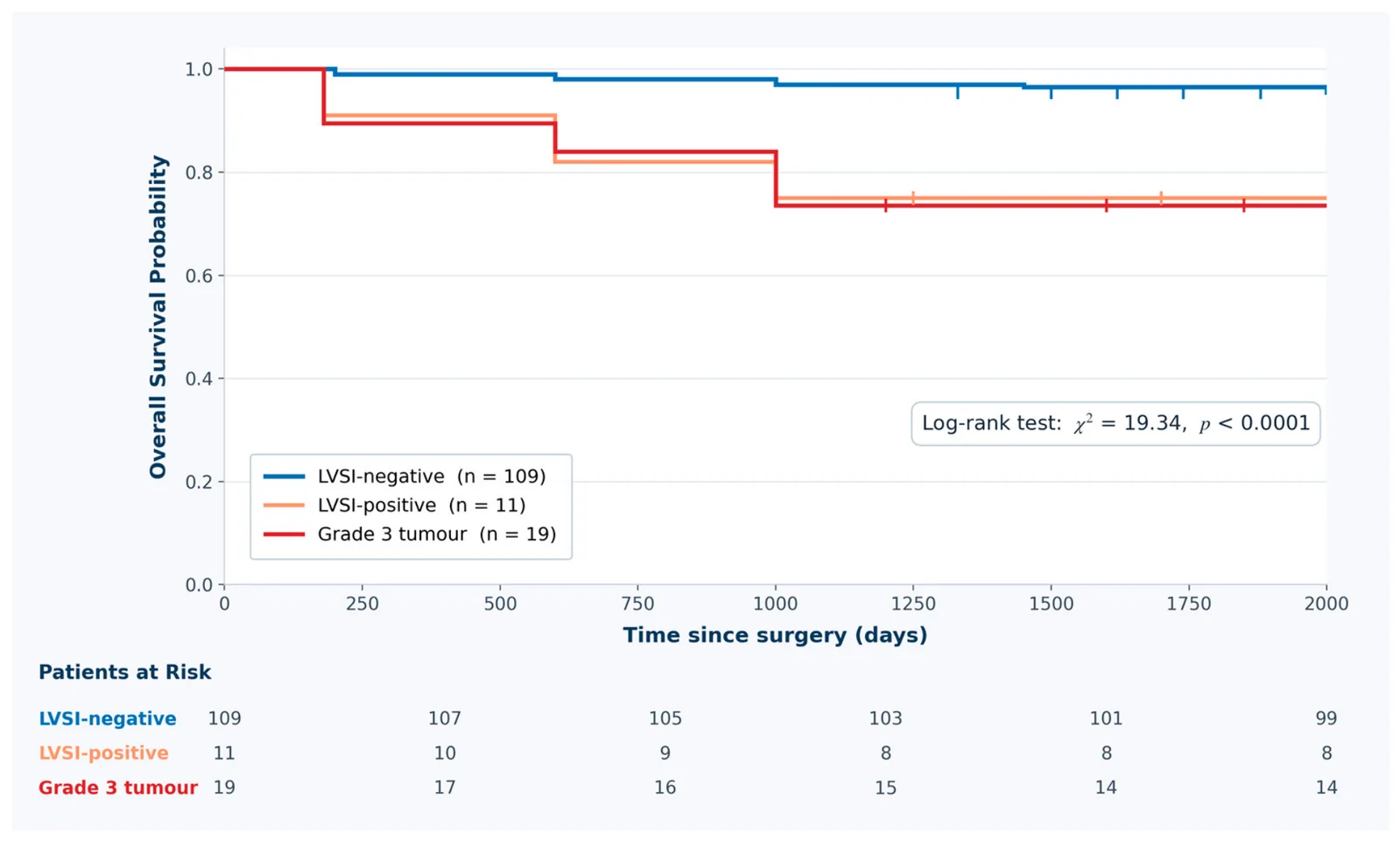
Kaplan–Meier Survival Curves by Clinical Groups. Kaplan–Meier estimates of overall survival stratified by clinical groups: LVSI-negative (blue, n = 109), LVSI-positive (orange, n = 11), and Grade 3 tumors (red, n = 19). The y-axis represents survival probability, and the x-axis shows time in days from diagnosis. Numbers at risk are displayed below the graph. Log-rank test: χ^2^ = 19.34, *p* < 0.0001. LVSI, lymphovascular space invasion.

Patients with LVSI-negative tumors had the most favorable survival profile, whereas those with LVSI-positive tumors or grade 3 disease experienced poorer outcomes. Kaplan–Meier analysis demonstrated clear separation among the clinical groups, as illustrated in Figure 1 (log-rank χ^2^ = 19.34, *p* < 0.0001), supporting the prognostic value of conventional clinicopathological stratification.

### 3.2. Individual Biomarker Associations with Survival

In univariate Cox regression analysis, summarized in Table 2, high expression of SIRT1, HMGB1, and Caspase-3 was significantly associated with poorer overall survival. The strongest individual associations were observed for HMGB1 (HR = 8.25, *p* < 0.001), SIRT1 (HR = 7.26, *p* < 0.001), and Caspase-3 (HR = 7.61, *p* = 0.008). Low Bcl-2 expression was significantly enriched in adverse clinicopathological groups in Table 1 and was therefore retained as a pre-specified adverse component of EIPS.

In marker-specific survival analysis, high Bcl-2 expression showed a nonsignificant protective association compared with low Bcl-2 expression (HR = 0.24, 95% CI: 0.05–1.09, *p* = 0.064), supporting the direction of low Bcl-2 as an adverse feature despite the limited number of events. Thus, Bcl-2 was retained because its biological direction, clinicopathological distribution, and near-threshold survival association were concordant with an adverse low-expression phenotype, even though its individual univariate *p*-value did not meet conventional statistical significance. Beclin-1 was evaluated descriptively and in marker-specific survival curves but was not included in the final EIPS. In contrast to Bcl-2, Beclin-1 did not show a significant survival association, did not demonstrate an adverse direction of effect in this cohort, and did not improve the interpretability or apparent performance of the final immunohistochemical prognostic score; therefore, it was excluded from EIPS. These findings supported construction of a prognostic score integrating complementary molecular pathways while acknowledging that some individual biomarker effects require cautious interpretation in a cohort with few events.

### 3.3. Multivariable Prognostic Modeling

**Figure 2:**
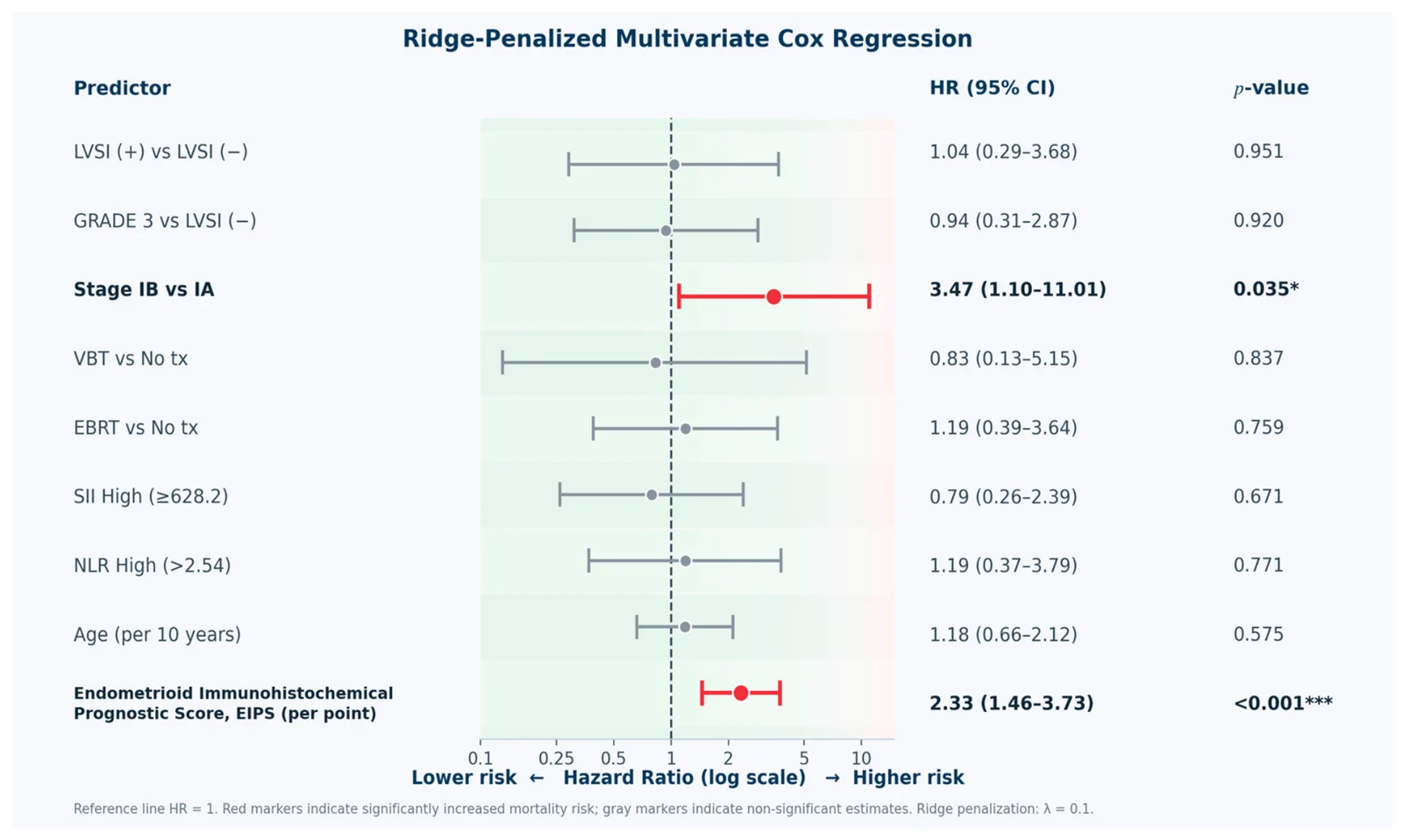
**Ridge-Penalized Multivariable Cox Regression Forest Plot.** Forest plot showing hazard ratios and 95% confidence intervals from the ridge-penalized multivariable Cox regression model used to reduce overfitting risk because of the limited number of survival events. The Endometrioid Immunohistochemical Prognostic Score (EIPS) remained independently associated with mortality (HR = 2.33, 95% CI: 1.46–3.73, *p* < 0.001), together with Stage IB disease (HR = 3.47, 95% CI: 1.10–11.01, *p* = 0.035). Variables positioned to the right of the reference line indicate increased mortality risk. * *p* < 0.05; *** *p* < 0.001.

Because the number of survival events was limited, ridge-penalized multivariable Cox regression was used to reduce overfitting risk. In this model, the Endometrioid Immunohistochemical Prognostic Score (EIPS) remained independently associated with mortality (HR = 2.33, 95% CI: 1.46–3.73, *p* < 0.001), indicating that the integrated biomarker profile provided prognostic information beyond conventional clinical variables. Stage IB disease also remained independently associated with poorer survival (HR = 3.47, 95% CI: 1.10–11.01, *p* = 0.035), whereas age, LVSI, grade, adjuvant treatment, SII, and NLR did not retain statistical significance after adjustment, as summarized in Figure 2. Among 137 complete cases with 12 deaths, adjusted hazard ratios increased across EIPS categories: intermediate-risk EIPS showed an adjusted HR of 2.67 (95% CI: 0.46–15.48, *p* = 0.273), and high-risk EIPS showed an adjusted HR of 6.99 (95% CI: 0.93–52.48, *p* = 0.059), both compared with low-risk EIPS.

### 3.4. Model Discrimination and Validation

**Figure 3:**
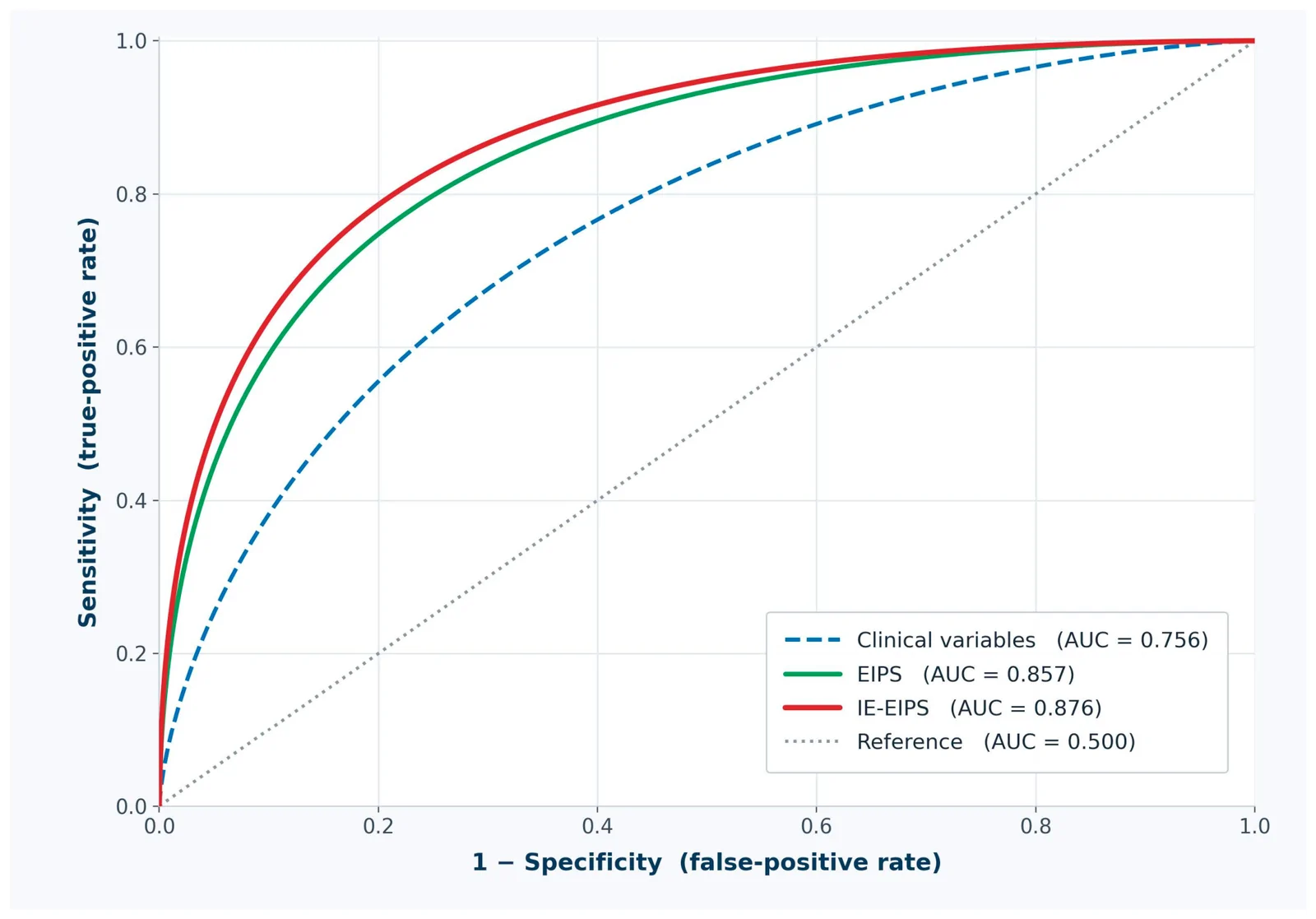
**ROC Curves Comparing the LVSI/Grade-Based Clinicopathologic Grouping, EIPS, and Ancillary IE-EIPS.** Receiver operating characteristic curves comparing mortality discrimination across prognostic approaches. The predefined LVSI/grade-based clinicopathologic grouping showed an AUC of 0.756, whereas the Endometrioid Immunohistochemical Prognostic Score (EIPS), the primary exploratory score of this study, showed a higher apparent AUC of 0.857. The Inflammatory-Enhanced Endometrioid Prognostic Score (IE-EIPS), evaluated as an ancillary exploratory model incorporating EIPS with the same clinicopathologic grouping and inflammatory variables, showed a modest additional apparent increase in discrimination (AUC = 0.876). The diagonal reference line represents no discrimination.

ROC analysis based on binary mortality status at the administrative censoring date demonstrated that the EIPS showed higher apparent discrimination for mortality compared with the predefined LVSI/grade-based clinicopathologic grouping, as summarized in Table 3 and illustrated in Figure 3.

The predefined LVSI/grade-based clinicopathologic grouping achieved an AUC of 0.756 (95% CI: 0.64–0.87), whereas EIPS showed an apparent AUC of 0.857 (95% CI: 0.78–0.93). This difference over the clinicopathologic grouping was statistically significant in bootstrap paired comparison (ΔAUC = 0.101, 95% CI: 0.02–0.18, *p* = 0.030). However, because only 12 deaths were observed, these discrimination estimates and internal validation results should be interpreted as exploratory stability analyses rather than definitive validation of predictive performance. The IE-EIPS, which incorporated EIPS together with the same clinicopathologic grouping and inflammatory indices, achieved the highest apparent AUC of 0.876 (95% CI: 0.80–0.95), with corrected and cross-validated AUC values of 0.871 and 0.868, but this larger score is particularly vulnerable to overfitting and should be considered hypothesis-generating only.

### 3.5. EIPS-Based Risk Stratification and Ancillary IE-EIPS Analysis

**Figure 4:**
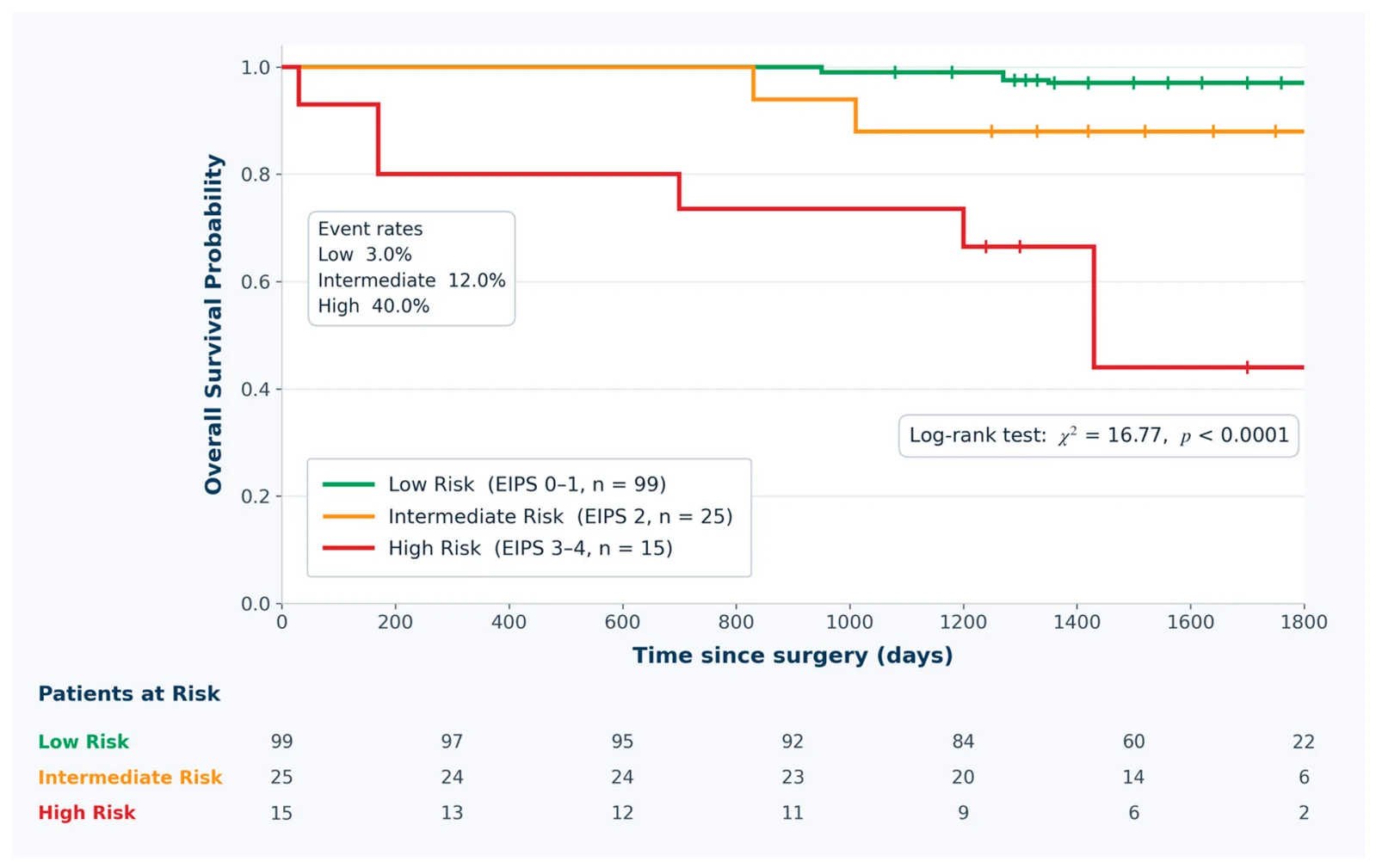
**Kaplan–Meier Curves by Endometrioid Immunohistochemical Prognostic Score (EIPS) Risk Groups.** Low-risk (EIPS 0–1, green, n = 99), intermediate-risk (EIPS 2, w, n = 25), and high-risk (EIPS 3–4, red, n = 15). The EIPS effectively discriminated survival outcomes with event rates of 3.0%, 12.0%, and 40.0% for low-, intermediate-, and high-risk groups, respectively. Log-rank test: χ^2^ = 16.72, *p* < 0.0001.

EIPS stratified patients into low-, intermediate-, and high-risk groups with increasing event rates and declining estimated 5-year overall survival, as summarized in Table 4 and illustrated in Figure 4. Event rates were 3.0% in the low-risk group (EIPS 0–1; 99/139), 12.0% in the intermediate-risk group (EIPS 2; 25/139), and 40.0% in the high-risk group (EIPS 3–4; 15/139), with corresponding estimated 5-year overall survival of approximately 97%, 88%, and 60%, respectively. Compared with low-risk EIPS, high-risk EIPS was associated with mortality in unadjusted time-to-event analysis (HR = 16.72, 95% CI: 4.18–66.91, *p* < 0.001), while intermediate-risk EIPS showed a less precise increase in risk (HR = 4.12, 95% CI: 0.83–20.43). In ridge-penalized multivariable analysis using the same categorical EIPS groups, adjusted hazard ratios were attenuated but remained clinically ordered: intermediate-risk EIPS HR = 2.67 (95% CI: 0.46–15.48, *p* = 0.273) and high-risk EIPS HR = 6.99 (95% CI: 0.93–52.48, *p* = 0.059), each compared with low-risk EIPS. Kaplan–Meier analysis confirmed separation among EIPS categories (log-rank χ^2^ = 16.72, *p* < 0.0001). IE-EIPS was retained as an ancillary exploratory model for assessing incremental prognostic information beyond EIPS.

### 3.6. Decision Curve Analysis of EIPS Model

**Figure 5:**
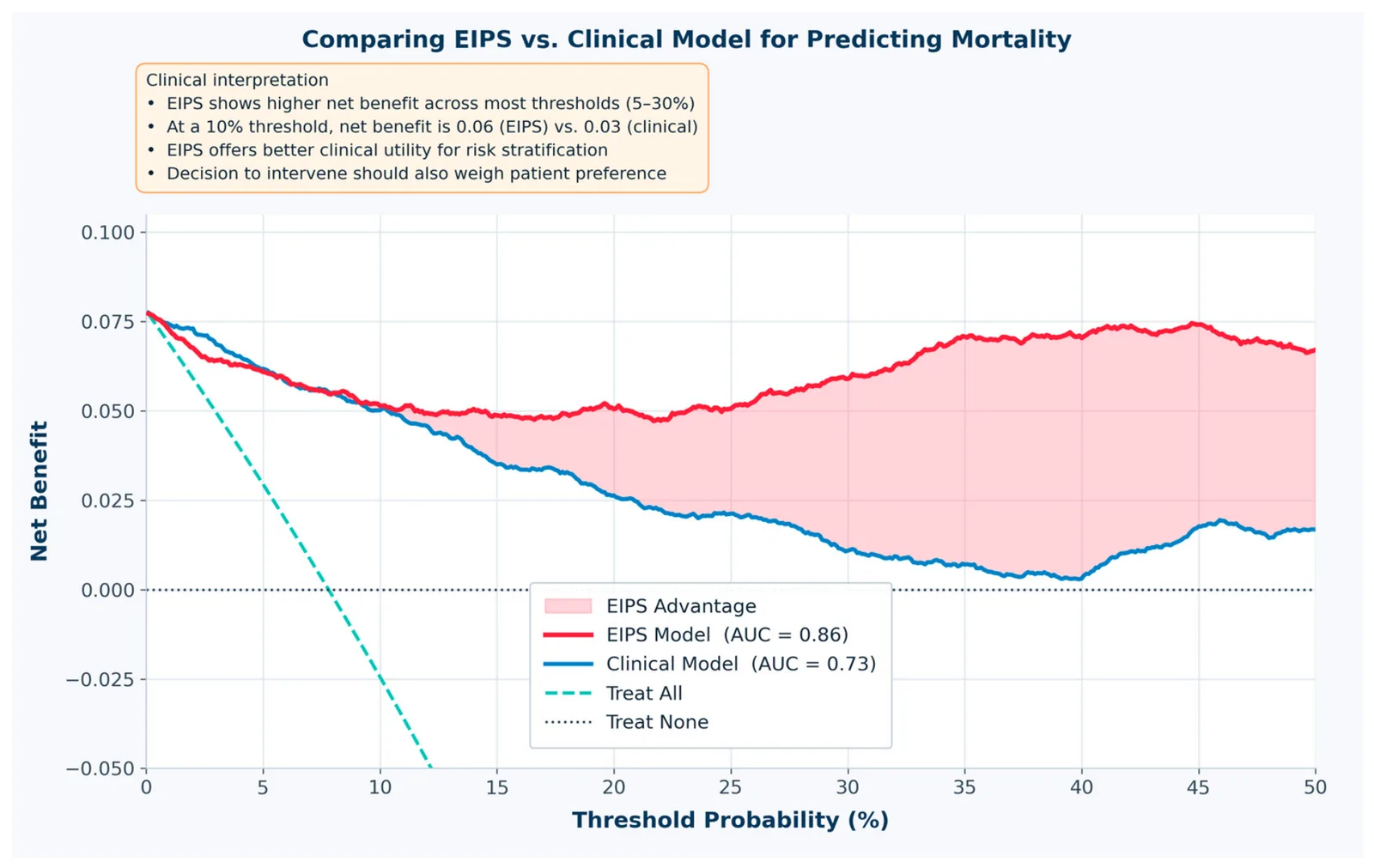
**Decision Curve Analysis of the EIPS Model.** Net benefit curves are shown across threshold probabilities of 5–30% for Treat All, Treat None, the predefined LVSI/grade-based clinical grouping, and the Endometrioid Immunohistochemical Prognostic Score (EIPS). EIPS showed higher net benefit than Treat All and clinical grouping across the evaluated range, suggesting exploratory utility for risk-adapted decision-making.

Decision curve analysis suggested possible exploratory clinical utility of EIPS across threshold probabilities of 5–30% (Figure 5). Decision curve analysis evaluates whether using a model would provide greater clinical net benefit than default strategies, such as treating all patients or treating no patients, across a range of threshold probabilities at which a clinician might consider intervention. Net benefit incorporates both true-positive and false-positive classifications and therefore reflects the trade-off between identifying patients at higher risk and avoiding unnecessary treatment or surveillance escalation. EIPS showed greater net benefit than both Treat All and clinical grouping across the evaluated threshold range, with net benefit ranging from 0.051 at 5% to 0.029 at 30%. In this analysis, the clinical grouping comparator refers to the predefined LVSI/grade-based clinical model used throughout the study. In contrast, the net benefit of clinical grouping decreased from 0.042 to 0.007 across the same threshold range, while Treat All showed lower or negative net benefit from the 20% threshold onward.

## 4. Discussion

In this study, we developed and evaluated an immunohistochemistry-based Endometrioid Immunohistochemical Prognostic Score (EIPS) integrating SIRT1, HMGB1, Bcl-2, and Caspase-3 expression in endometrioid-type endometrial cancer. The main finding was that EIPS was associated with overall survival, showed higher apparent mortality discrimination than the predefined LVSI/grade-based clinicopathologic grouping, and provided risk separation across low-, intermediate-, and high-risk categories. FIGO stage was evaluated separately to avoid conflating anatomic stage with the LVSI/grade comparator. These results support the concept that combined assessment of biologically complementary pathways may warrant further investigation alongside conventional guideline-based factors such as grade and lymphovascular space invasion, which remain central to endometrial cancer risk assessment [2]. Given that only 12 deaths occurred and that the comparator was not a formal molecular ESGO/ESTRO/ESP risk classification, these findings should be interpreted as exploratory and hypothesis-generating rather than as evidence of a validated clinical prediction model.

The prognostic role of SIRT1 in endometrial cancer remains biologically plausible but context-dependent. Experimental work has shown that SIRT1 may promote endometrial carcinoma cell proliferation, tumor growth, autophagy, and resistance to cisplatin and paclitaxel, supporting its potential contribution to aggressive tumor behavior [5,6]. Other clinical studies have reported variable associations between SIRT1 expression and outcome, including findings suggesting improved progression-free survival in mixed uterine cancer cohorts or no independent prognostic effect in non-endometrioid disease [7,8]. These differences may reflect variation in histological subtype, subcellular staining pattern, scoring method, and clinical composition. In the present endometrioid-only cohort, high SIRT1 expression was associated with poorer survival and contributed substantially to EIPS, suggesting that SIRT1 may be most informative when interpreted as part of an integrated molecular risk profile rather than as an isolated marker.

HMGB1 also has a complex role in tumor biology. As a nuclear protein that can be released extracellularly under cellular stress, HMGB1 may function as a damage-associated molecular pattern and promote inflammation, autophagy, invasion, angiogenesis, and therapy resistance [9,10]. Prior work has linked HMGB1-mediated autophagy to cancer progression and drug resistance, while endometrial cancer studies have reported both tumor-promoting and potentially tumor-suppressive effects depending on model system and histological context [11,12]. In our cohort, high HMGB1 expression was strongly associated with poorer overall survival in univariate analysis and formed a key component of EIPS. This supports the interpretation that HMGB1 may reflect an inflammatory and stress-adaptive tumor phenotype that is clinically relevant when combined with other pathway markers.

Beclin-1 was evaluated descriptively and in marker-specific survival analyses but was not included in the final EIPS. This decision is consistent with the dual and context-dependent role of autophagy in cancer. Beclin-1-mediated autophagy may suppress early tumorigenesis by maintaining cellular homeostasis, yet in established tumors it may support survival under hypoxia, nutrient deprivation, and treatment-related stress [13,14]. Previous studies in endometrial adenocarcinoma have reported that high Beclin-1 expression may define poorer prognosis, whereas broader autophagy literature emphasizes that its impact depends on tumor stage and biological context [15,16]. In this study, Beclin-1 did not strengthen the final immunohistochemical model, suggesting that its prognostic contribution may be less direct or more context-dependent than the markers retained in EIPS.

The inclusion of Bcl-2 and Caspase-3 in EIPS reflects dysregulation of apoptotic balance in endometrial carcinogenesis. Low Bcl-2 expression and altered Bcl-2/Bax balance have been associated with adverse clinicopathological features in endometrial carcinoma, whereas Caspase-3 reflects activation of the execution phase of apoptosis [17,18,19]. Although Caspase-3 is traditionally considered a cell-death marker, high tumor expression may reflect increased apoptotic turnover, biological instability, and aggressive tumor dynamics rather than effective tumor suppression; shorter survival has also been reported in tumors with high caspase-3 expression [18,19]. In the present analysis, high Caspase-3 expression was significantly associated with poorer survival. Low Bcl-2 expression was strongly associated with adverse clinicopathological grouping, and marker-specific Cox analysis showed that high Bcl-2 expression was associated with lower mortality risk compared with low Bcl-2 expression, although this individual association was not statistically significant. We therefore retained low Bcl-2 in EIPS because the direction of effect was biologically consistent, its distribution was strongly linked to adverse clinicopathological features, and its borderline survival association may have been underpowered because only 12 deaths occurred. By comparison, Beclin-1 was excluded because its association with survival was weak and directionally non-adverse in this cohort, making its inclusion less justified statistically and biologically. Therefore, low Bcl-2 expression was retained as a biologically plausible adverse component of EIPS and is best interpreted within the composite apoptotic profile rather than as a standalone survival marker.

The EIPS showed higher apparent discrimination than the LVSI/grade-based clinicopathologic grouping in ROC analysis and was associated with mortality in ridge-penalized multivariable modeling. This is relevant because conventional risk assessment based on grade, stage, myometrial invasion, and LVSI remains clinically useful but imperfect, particularly among early stage endometrioid tumors with heterogeneous outcomes [2]. In the present study, the LVSI/grade grouping functioned as an interpretable comparator built from available variables, not as a substitute for formal guideline-based molecular risk stratification. Recent biomarker literature emphasizes the need for practical, reproducible, and clinically interpretable panels rather than reliance on single markers alone [25]. However, because EIPS combines four biomarkers and was evaluated in only 12 events, its apparent performance may be inflated by overfitting despite penalized modeling and internal resampling. EIPS therefore should be viewed as a simple exploratory immunohistochemical score that requires independent validation before being considered for clinical use.

The ancillary Inflammatory-Enhanced Endometrioid Prognostic Score (IE-EIPS), which added the LVSI/grade-based clinical grouping and inflammatory indices to EIPS, produced only a modest apparent improvement in discrimination. This suggests that most prognostic information may already have been captured by EIPS, while systemic inflammation and available clinicopathological features may provide limited incremental value in this cohort. Importantly, the clinical component of IE-EIPS should be interpreted as a transparent clinicopathologic comparator rather than as a formal molecular risk group. The inclusion of blood-based inflammatory parameters in IE-EIPS was supported by emerging evidence that composite immune-inflammatory indices may have prognostic value in endometrial cancer. In particular, Chalabıyev et al. evaluated the pan-immune-inflammation value in endometrial cancer patients undergoing adjuvant therapy and reported that higher PIV was associated with adverse clinicopathological features and poorer survival, including increased mortality risk above a cutoff of 350 [26]. Although IE-EIPS used SII and NLR rather than PIV because monocyte-count data were not incorporated into the present score, these findings provide relevant context for the broader concept that systemic inflammatory status may add prognostic information to tumor-based risk markers.

Decision curve analysis suggested possible clinical usefulness of EIPS itself, showing greater net benefit than Treat All or clinical grouping across the evaluated threshold probability range. This approach is valuable because it evaluates whether a prediction model offers potential clinical benefit across decision thresholds, not only whether it improves statistical discrimination [27,28]. Nevertheless, no inference about clinical implementation should be drawn until the score is externally validated in larger multicenter cohorts with adequate event numbers.

From an implementation perspective, EIPS should be introduced cautiously and initially evaluated in academic or referral centers with gynecologic oncology, pathology, laboratory quality assurance, and data-analysis expertise. Although the score is based on immunohistochemistry rather than sequencing, reliable use would still require standardized tissue handling, validated staining and scoring protocols, trained personnel, and ongoing quality control. Community hospitals and smaller healthcare systems may face barriers related to equipment, testing volume, training, and cost-effectiveness; therefore, if external validation confirms clinical utility, broader access may be best supported through centralized reference laboratories or regional testing hubs, including national or government-supported models where appropriate.

This study has several strengths. The cohort was restricted to endometrioid-type endometrial carcinoma, reducing histological heterogeneity. This focus was intentional because endometrioid carcinoma represents the most common subtype but remains clinically heterogeneous, whereas non-endometrioid tumors such as serous, clear cell, carcinosarcoma, undifferentiated/dedifferentiated carcinoma, and mixed tumors have distinct biology and should be evaluated separately [2,3,4].

The biomarker panel was selected to represent biologically relevant and interacting pathway-related signals, including stress adaptation, inflammatory signaling, autophagy regulation, and apoptotic balance. The rationale for selecting SIRT1, HMGB1, Beclin-1, Bcl-2, and Caspase-3 was therefore to examine a feasible routine-IHC panel spanning complementary tumor-survival, inflammatory, autophagy-related, and apoptosis-related biology, rather than to claim comprehensive pathway measurement. However, these markers should be interpreted as selected surrogates rather than comprehensive measures of apoptosis, pyroptosis, or autophagy, and the present study does not claim that a single marker or limited marker pair can fully define those biological processes. In addition, the analysis combined survival modeling, ROC-based mortality discrimination, exploratory internal validation, and decision curve analysis, providing complementary exploratory evidence for both statistical performance and possible clinical usefulness. However, these strengths do not overcome the limited events-per-variable problem, and all model-based findings should be considered preliminary.

Several limitations should also be acknowledged. The retrospective single-center design may limit generalizability, and the number of deaths was small, increasing the risk of model instability and wide confidence intervals. Because the cohort was deliberately limited to pure stage I endometrioid-type endometrial carcinoma, the findings cannot be generalized to non-endometrioid or mixed histologic subtypes without separate validation [2,3,4]. Although ridge-penalized regression and internal validation were used to reduce overfitting, these approaches cannot replace external validation.

The immunohistochemical cutoffs were selected to be practical and reproducible, but alternative scoring systems, digital quantification, or compartment-specific assessment may yield different results. Although two pathologists reviewed the staining using a predefined scoring approach and consensus review was used for discrepant cases, formal interobserver agreement statistics such as kappa could not be calculated retrospectively; this should be considered a reproducibility limitation, and future validation studies should prospectively report interobserver agreement.

Finally, TCGA-derived molecular classification variables, including POLE mutation, mismatch repair status, p53 abnormality, and NSMP status, were not incorporated [3]. This is important because contemporary guidelines and the 2023 FIGO staging framework increasingly emphasize tumor biology in endometrial cancer risk stratification [2,4]. These subgroups refine prognosis and guide adjuvant treatment beyond traditional histopathological parameters [3,4], and integrating POLE, MMR, and p53 status improves prognostic precision and aligns risk assessment with targeted and immunotherapeutic approaches [29]. Although molecular data were unavailable, EIPS markers reflecting stress adaptation, inflammatory signaling, and apoptotic balance may serve as a pragmatic adjunct when molecular testing is unavailable, delayed, or resource-limited. Thus, EIPS should complement, not replace, molecular classification, and future studies combining both approaches may yield more comprehensive prognostic models.

Molecular classification can substantially modify prognosis: POLE-mutated tumors generally have favorable outcomes, p53-abnormal tumors poor outcomes, and mismatch repair-deficient tumors distinct prognostic and therapeutic implications, and NSMP tumors often require further clinicopathological stratification [3,4]. EIPS should be viewed as an exploratory IHC-based adjunct for settings where full molecular classification is unavailable or delayed, not as an alternative to molecular staging or guideline-based risk assignment. Future studies should assess whether EIPS adds prognostic value within contemporary molecular risk groups.

Overall, these findings suggest that EIPS may capture a biologically coherent adverse-risk phenotype characterized by SIRT1-mediated stress adaptation, HMGB1-related inflammatory signaling, apoptosis-related imbalance, and loss of Bcl-2-associated differentiation signals. By integrating these features into a single immunohistochemistry-based score, EIPS may complement established clinicopathological risk stratification in early stage endometrioid-type endometrial cancer. In contemporary practice, however, any proposed IHC-based score must be interpreted within the molecular framework of endometrial cancer, including POLE, MMR, p53, and NSMP status, because these subgroups influence prognosis and treatment selection. The present data therefore support only exploratory prognostic stratification and suggest a possible role for EIPS as a low-cost adjunct in resource-limited settings, not as a replacement for molecular classification or validated guideline-based risk groups. Larger multicenter studies with molecular classification, standardized staining protocols, adequate numbers of outcome events, and longer follow-up are needed to confirm its reproducibility and determine whether EIPS can guide individualized adjuvant treatment or surveillance strategies.

## 5. Conclusions

This study suggests that an Endometrioid Immunohistochemical Prognostic Score (EIPS) based on SIRT1, HMGB1, Bcl-2, and Caspase-3 expression may provide exploratory prognostic stratification in this cohort of patients with endometrioid-type endometrial cancer. EIPS was associated with overall survival and showed higher apparent discriminatory performance than the predefined LVSI/grade-based clinicopathologic grouping, supporting its potential value as an adjunctive risk-stratification tool. IE-EIPS, which incorporated EIPS with the same clinicopathologic grouping and inflammatory indices, offered additional ancillary prognostic information, but both scores should be interpreted as exploratory. Neither EIPS nor IE-EIPS can be considered reliably validated in this cohort, and prospective multicenter external validation with adequate event numbers and contemporary molecular classification is needed before clinical implementation.

## Figures and Tables

**Table 1:** Baseline Characteristics of Study Population.

Variable	Total (n = 139)	LVSI-Negative (n = 109)	LVSI-Positive (n = 11)	Grade 3 (n = 19)	*p* Value
**Demographics**					
Age, years (mean ± SD)	61.2 ± 10.8	60.8 ± 10.5	59.5 ± 12.1	64.2 ± 11.4	0.412
Age < 60 years, n (%)	52 (37.4%)	42 (38.5%)	5 (45.5%)	5 (26.3%)	0.438
**Clinical Features**					
Stage IA, n (%)	101 (72.7%)	89 (81.7%)	3 (27.3%)	9 (47.4%)	<0.001 ***
Stage IB, n (%)	38 (27.3%)	20 (18.3%)	8 (72.7%)	10 (52.6%)	—
Adjuvant Treatment, n (%)	42 (30.2%)	25 (22.9%)	7 (63.6%)	10 (52.6%)	0.001 **
**Follow-up and Outcomes**					
Follow-up, days (median, IQR)	1577 (1498–1677)	1583 (1513–1717)	1498 (1290–1606)	1528 (1252–1603)	0.089
Deaths, n (%)	12 (8.6%)	4 (3.7%)	3 (27.3%)	5 (26.3%)	<0.001 ***
**IHC Marker Expression**					
SIRT1 High (score 2–3), n (%)	22 (15.8%)	14 (12.8%)	3 (27.3%)	5 (26.3%)	0.178
HMGB1 High (score 2–3), n (%)	16 (11.5%)	10 (9.2%)	2 (18.2%)	4 (21.1%)	0.237
Beclin-1 High (score 2–3), n (%)	56 (40.3%)	45 (41.3%)	4 (36.4%)	7 (36.8%)	0.892
Bcl-2 Low (score 0–1), n (%)	46 (33.1%)	24 (22.0%)	6 (54.5%)	16 (84.2%)	<0.001 ***
Caspase-3 High (score 2–3), n (%)	50 (36.0%)	36 (33.0%)	5 (45.5%)	9 (47.4%)	0.357
**Inflammatory Indices**					
SII (median, IQR)	6292 (4336–8362)	6093 (4172–8346)	7209 (6202–9057)	7867 (5762–10694)	0.156
NLR (median, IQR)	2.14 (1.65–2.80)	2.10 (1.60–2.70)	2.40 (1.70–3.70)	2.60 (2.10–3.50)	0.089

Abbreviations: LVSI, lymphovascular space invasion; IHC, immunohistochemistry; SII, systemic immune-inflammation index; NLR, neutrophil-to-lymphocyte ratio. ** *p* < 0.01, *** *p* < 0.001.

**Table 2:** Univariate Cox Regression Analysis for Overall Survival.

Variable	*Events/n*	*HR*	*95% CI*	*p-Value*
**Clinical Groups**				
LVSI negative	4/109	1.00 (ref)	—	—
LVSI positive	3/11	9.87	2.21–44.12	0.003 **
Grade 3	5/19	9.08	2.56–32.21	<0.001 ***
**Clinical Variables**				
Age (per year)	—	1.03	0.98–1.08	0.254
Stage IB vs. IA	5/37 vs. 7/102	2.91	0.93–9.08	0.066
Adjuvant Treatment (yes vs. no)	5/42 vs. 7/97	1.82	0.58–5.71	0.307
**IHC Markers**				
SIRT1 High vs. Low	7/22 vs. 5/117	7.26	2.29–22.99	<0.001 ***
HMGB1 High vs. Low	6/16 vs. 6/123	8.25	2.51–27.13	<0.001 ***
Beclin-1 High vs. Low	3/56 vs. 9/83	0.45	0.12–1.65	0.227
Bcl-2 High vs. Low	4/93 vs. 8/46	0.24	0.05–1.09	0.064
Caspase-3 High vs. Low	8/50 vs. 4/89	7.61	1.69–34.21	0.008 **
**Inflammatory Indices**				
SII ≥ median vs. <median	9/70 vs. 3/69	2.89	0.91–9.16	0.071
NLR ≥ median vs. <median	9/70 vs. 3/69	3.47	0.95–12.70	0.060
**Endometrioid Immunohistochemical Prognostic Score (EIPS; per point)**	—	2.45	1.68–3.57	<0.001 ***

Abbreviations: HR, hazard ratio; CI, confidence interval; IHC, immunohistochemistry; LVSI, lymphovascular space invasion; SII, systemic immune-inflammation index; NLR, neutrophil-to-lymphocyte ratio. ** *p* < 0.01, *** *p* < 0.001.

**Table 3:** Prognostic Model Performance and Validation.

Variable	AUC	95% CI	Corrected AUC	CV AUC (5-Fold)
**Model**				
**LVSI/Grade-Based Clinicopathologic Grouping**	**0.756**	**0.64–0.87**	—	—
Endometrioid Immunohistochemical Prognostic Score (EIPS)	0.857	0.78–0.93	0.863	0.862
Inflammatory-Enhanced Endometrioid Prognostic Score (IE-EIPS)	0.876	0.80–0.95	0.871	0.868
**Model Comparison (Bootstrap Paired Test, n = 1000 iterations)**				
Comparison	**ΔAUC**	**95% CI**	***p*** **-value**	
EIPS vs. Clinical Groups	0.101	0.02–0.18	0.030 *	
IE-EIPS vs. Clinical Groups	0.120	0.03–0.21	0.010 *	

Abbreviations: AUC, area under the receiver operating characteristic curve; CI, confidence interval; EIPS, Endometrioid Immunohistochemical Prognostic Score; CV, cross-validation; IE-EIPS, Inflammatory-Enhanced Endometrioid Prognostic Score; LVSI, lymphovascular space invasion. * *p* < 0.05.

**Table 4:** Risk Stratification by Endometrioid Immunohistochemical Prognostic Score.

Risk Group	EIPS Score	n (%)	Events/Event Rate	Estimated 5-Year OS	HR (95% CI)
**Low Risk**	0–1	99 (71.2%)	3/3.0%	~97%	1.00 (ref)
**Intermediate Risk**	2	25 (18.0%)	3/12.0%	~88%	4.12 (0.83–20.43)
**High Risk**	3–4	15 (10.8%)	6/40.0%	~60%	16.72 (4.18–66.91) ***
***Log-rank test: χ^2^ = 16.72, p < 0.0001.***					

Abbreviations: EIPS, Endometrioid Immunohistochemical Prognostic Score; HR, hazard ratio; CI, confidence interval; OS, overall survival. EIPS = SIRT1 High + HMGB1 High + Caspase-3 High + low Bcl-2. Score range 0–4. *** *p* < 0.001.

## Data Availability

The data that support the findings of this study are available from the corresponding author upon reasonable request.

## Supplementary Materials

The following supporting information can be downloaded at: https://www.mdpi.com/article/10.3390/diagnostics16172701/s1.

## Abbreviations

The following abbreviations are used in this manuscript: AUC, area under the receiver operating characteristic curve; CI, confidence interval; EIPS, Endometrioid Immunohistochemical Prognostic Score; CV, cross-validation; IE-EIPS, Inflammatory-Enhanced Endometrioid Prognostic Score; FIGO, International Federation of Gynecology and Obstetrics; HMGB1, high mobility group box 1; HR, hazard ratio; IHC, immunohistochemistry; IQR, interquartile range; LVSI, lymphovascular space invasion; NAD^+^, nicotinamide adenine dinucleotide; NLR, neutrophil-to-lymphocyte ratio; NSMP, no specific molecular profile; OS, overall survival; POLE, DNA polymerase epsilon catalytic subunit; ROC, receiver operating characteristic; SD, standard deviation; SII, systemic immune-inflammation index; SIRT1, sirtuin 1.
