# Supplementary material for: Endometrioid Immunohistochemical Prognostic Score Integrating SIRT1, HMGB1, Bcl-2, and Caspase-3 Expression in Endometrioid-Type Endometrial Cancer
Source: Diagnostics (Basel). 2026 Aug 24;16(17):2701. doi: 10.3390/diagnostics16172701 (PMC13565476; doi:10.3390/diagnostics16172701)
Supplement: Supplementary file 1 [file diagnostics-16-02701-s001.zip › diagnostics-4427132-supplementary.pdf]

Supplementary Materials

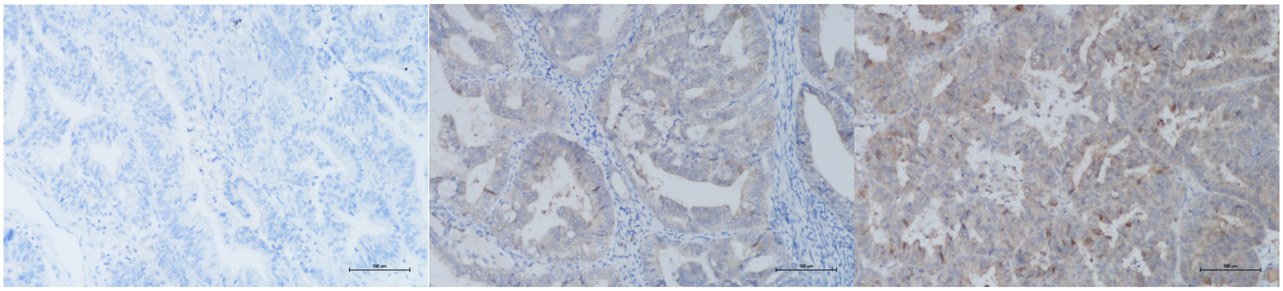

Supplementary Figures S1–S3. *Representative SIRT1 immunostaining: left, 0; middle, 1+; right, 2+; ×200.*

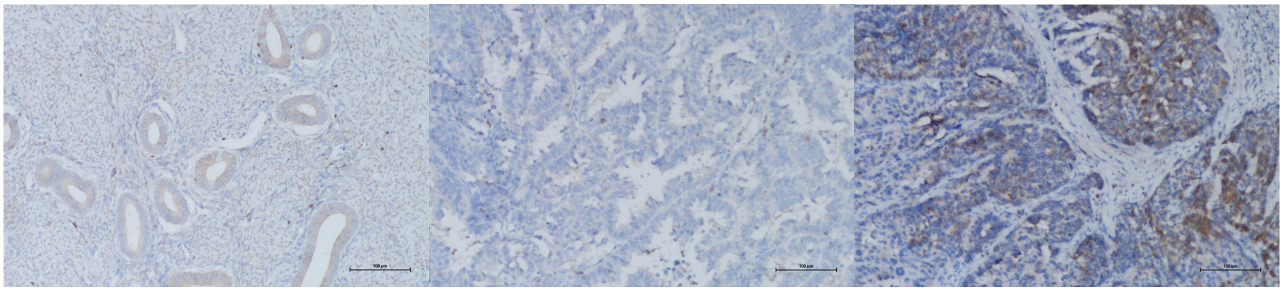

Supplementary Figures S4–S6. *Representative HMGB1 immunostaining: left, 0; middle, 1+; right, 2+; ×200.*

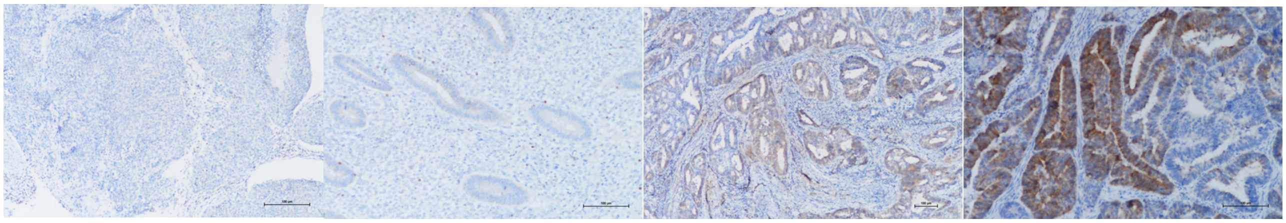

Supplementary Figures S7–S10. *Representative Beclin-1 immunostaining: left, 0; middle left, 1+; middle right, 2+; right, 3+; ×200.*

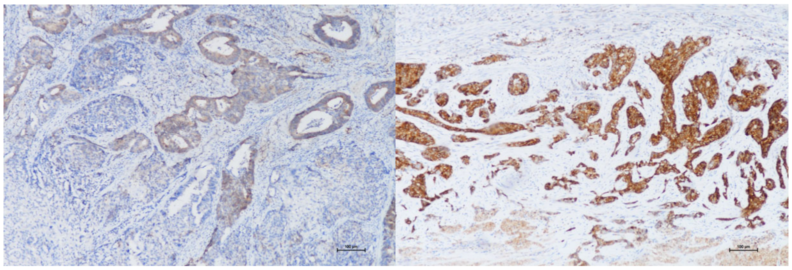

Supplementary Figures S11 and S12. *Representative Caspase-3 immunostaining: left, 2+; right, 3+; ×200.*

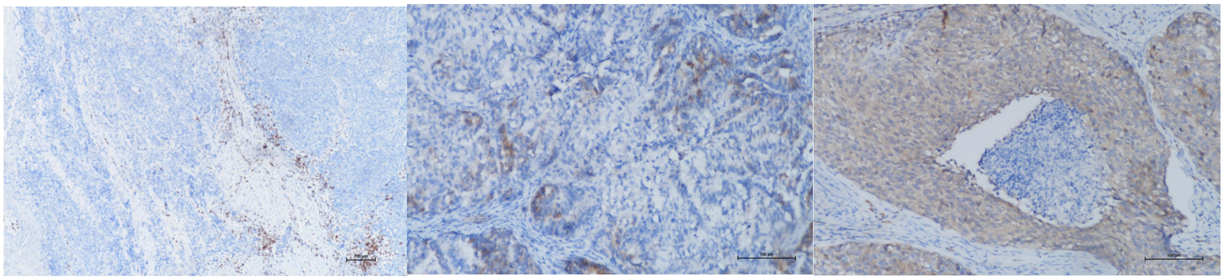

Supplementary Figures S13–S15. *Representative Bcl-2 immunostaining: left, 0; middle, 1+; right, 2+; ×200.*
